# Angiotensin-converting enzyme gene insertion/deletion polymorphism is not associated with BMI in Korean adults

**DOI:** 10.20463/pan.2020.0005

**Published:** 2020-03-31

**Authors:** Insu Kwon

**Affiliations:** 1 Research Institute of Sports Science & Industry, Hanyang University, Seoul Republic of Korea

**Keywords:** angiotensin-converting enzyme (ACE), gene polymorphism, overweight, obesity, BMI, blood pressure

## Abstract

**[Purpose]:**

Recent studies have demonstrated a probable association between *ACE* I/D polymorphism and obesity. Thus, this study aimed to investigate whether *ACE* I/D polymorphism influenced the susceptibly of developing obesity in Korean adults.

**[Methods]:**

A total of 353 healthy Korean adults aged between 30 and 82 years were recruited, including 157 males and 196 females. Among the participants, 103 (29.2 %) were classified as normal (BMI < 23 kg/m^2^), 117 (33.1 %) as overweight (23 kg/m^2^ ≤ BMI < 25 kg/m^2^), and 133 (37.7 %) as obese (BMI ≥ 25 kg/m^2^). ACE polymorphism (rs1799752) analysis was performed using the MGB TaqMan® SNP Genotyping assay with 3 types of primers and 2 types of probes. The distributions of the ACE genotypes and allele frequencies were analyzed among the three groups using the Hardy-Weinberg equilibrium, chi-square tests, and multiple regression analysis.

**[Results]:**

The distribution of the *ACE* genotypes were as follows: normal [II: n=38 (36.9 %), ID: n=46 (36.8 %), DD: n=19 (18.4 %)], overweight [II: n=43 (36.8 %), ID: n=55 (47.0 %), DD: n=19 (16.2 %)], and obese [II: n=41 (30.8 %), ID: n=76 (57.0 %), DD: n=16 (12.0 %)]. Unexpectedly, the I allele, rather than the D allele, was common in the obese group.

**[Conclusion]:**

*ACE* I/D polymorphism is not associated with BMI in Korean adults. Thus, it is unlikely to be a powerful candidate gene for obesity in Korean adults.

## INTRODUCTION

Obesity, defined as an abnormal or excessive accumulation of body fat, has become a global pandemic with a high prevalence in both adults and children^1^. Obesity is a direct and/or indirect risk factor for metabolic syndrome, a collection of pathological conditions comprising of abdominal obesity, dyslipidemia, hyperglycemia and hypertension^2^. Moreover, it is associated with a greater burden of disease, including a continuous increase in morbidity and mortality^3^. The etiological determinants and pathophysiological mechanisms for obesity have not yet been clearly elucidated; However, the susceptibility for developing an obese phenotype is governed by both genetic and environmental factors. Genetic factors or more specifically, obesity-related genes, are targeted for prevention strategies^4,5^, whereas treatment strategies rely on environmental factors including excessive food intake, physical inactivity, and stress^6,7^. Notably, obesogenic effects of candidate gene polymorphisms have been recently demonstrated^4,5,8^.

Renin angiotensin system (RAS) is a complicated hormonal cascade that mediates endocrine, paracrine, and intracrine actions. The classical ‘circulating RAS’ regulates the blood pressure and extracellular fluid volume whereas, the local ‘tissue RAS’ promotes the growth and development of most organs and tissues^9,10^. In the canonical pathway, a series of domino effects are triggered by renin, a key component of RAS that is released by the kidney. Renin cleaves the angiotensinogen (AGT) produced by the liver to angiotensin I (Ang I) which is subsequently converted to angiotensin II (Ang II) by angiotensin-converting enzyme (ACE)^11^. Ang II is activated by interaction with Ang II type 1 receptor (AT1) and can thereafter act as a potent vasoconstrictor to directly induce high blood pressure. The Ang II also indirectly mediates an increase in plasma volume through sodium and water reabsorption by stimulating the secretion of aldosterone, a hormone produced in the adrenal cortex^12^. Importantly, AGT, ACE, and Ang II expression have been observed in human adipose tissue^13-15^.

The major gene polymorphism of *angiotensin-converting enzyme (ACE)* is characterized by the presence (insertion: I) or absence (deletion: D) of a 287 base-pair fragment within intron 16. This leads to genetic variances as evident in the II, ID, and DD genotypes^16^. Additionally, these genetic variants of *ACE* are associated with different serum concentration of *ACE*^17^. Moreover, the D allele of *ACE* is likely to be involved in several chronic diseases including coronary heart disease^18^, diabetic nephropathy^19^, hypertension^20^, as well as obesity^21^. Conversely, the findings regarding the dominant effect of *ACE* I/D polymorphism on obesity are inconsistent as no significant association was found in the parallel studies conducted on a cluster of Asian (Korean adult women^22,23^ and children^5,24^), Chinese^25,26^, Japanese^27^, Caucasian^21,28,29^ and even African^30^ populations. No studies have been conducted evaluating the association between the *ACE* I/D polymorphism and obesity in Korean adults without the population sample being stratified on the basis of sex (male/female) and age (young/elderly), even though this association should be explored within the ‘*population genetics*’ field. Therefore, this study was investigated whether the *ACE* I/D polymorphism affects the susceptibility of Korean adults to develop obesity, regardless of sex or age.

## METHODS

### Participants

Healthy Korean adults aged between 30-82 years were recruited, including 157 males and 196 females. The experimental procedure was approved by the Institutional Review Board; the 353 participants were provided with a basic explanation of the process and purpose of the study prior to obtaining their written consent before commencement of the study.

### Anthropometric indexes and blood pressure

Height (cm) and body weight (kg) were measured to calculate the body mass index (BMI, kg/m^2^), a measure of the body weight divided by the square of height, that served as the diagnostic criteria to distinguish between the normal and obese individuals. The criteria for categorization of the BMI of Korean adults was adopted in accordance with the 2018 guideline for the management of obesity that was provided by the Korean Society for the Study of Obesity (KSSO)^31^. A few parameters for anthropometric measurements including height, body weight, and percent body fat (PBF) were evaluated by bioelectrical impedance analysis (InBody 720, Korea). A BMI greater than 23 kg/m^2^ and below 25 kg/m^2^ was classified as overweight and a BMI greater than or equal to 25 kg/m^2^ was categorized as obese. Blood pressure (BP) was assessed by both systolic (SBP) and diastolic (DBP) blood pressures. The subjects remained stable in the sitting upright position for a minimum of 10 minutes prior to their pressure being measured on their dominant arm using a standard clinical sphygmomanometer. Hypertension was defined as SBP and DBP equal or greater to 140 mmHg and 90 mmHg, respectively.

### DNA extraction and ACE I/D genotype

Genomic DNA was isolated from whole blood cells using Puregene^^®^^ DNA Purification Kit (Cat. #D550, Gentra, USA). Firstly, 5-15 μl of gDNA was extracted from 300 μl of whole blood sample. The *ACE* polymorphism (rs1799752) analysis was performed using the MGB TaqMan^®^ SNP Genotyping assay with 3 types of primers and 2 types of probes^32^. The nucleotide sequences utilized in the study are as follows: Primer *ACE*111: 5’-CCC-ATC-CTT-TCT-CCC-ATT-TCT-C-3’, Primer *ACE*112: 5’-AGC-TGG-AAT-AAA-ATT-GGC-GAA-AC-3’, Primer *ACE*113: 5’-CCT-CCC-AAA-GTG-CTG-GGA-TTA-3’, the I-allele-specific sequence: VIC-5’AGG-CGT-GAT-ACA-GTC-A-3’-MGB, and the D-allele-specific sequence: FAM-5’TGC-TGC-CTA-TAC-AGT-CA-3’MGB. The reaction components necessary for the amplification mix were 10-50 ng gDNA, TaqMan^®^ Genotyping Master Mix, 10 pmol/each primer, 150 nM VIC probe, and 75 nM FAM probe. Finally, target gene amplification was performed using the ABI 7900HT Real-Time PCR system (Applied Biosystems^®^, USA). The cycling profile included the initial denaturation at 92 ℃ for 15 seconds, followed by 40 cycles of annealing and extension at 57 ℃ for 1 minute. The calculation for the genotypes was performed automatically (SDS Plate Utility v2.2 software, Applied Biosystems^®^, USA).

### Statistical analysis

The *ACE* genotype and allele frequencies were obtained by direct count. The Hardy-Weinberg equilibrium for genotype distribution of each group was then estimated by the chi-square test. Differences in the allele frequencies and genotypes for the normal and overweight/obese groups were compared using chi-square tests (2×2 for the two groups and alleles, and 2×3 for the two groups and genotypes). Differences in the allele frequencies and genotypes between the normal, overweight, and obese groups were also analyzed using chi-square tests (3×2 for the three groups and alleles, 3×3 for the three groups and genotypes). Multiple comparisons for the differences between the two group were performed when the chi-square test showed significant differences in the 3×2 tables for the three groups and alleles and the 3×3 tables for the three groups and genotypes. According to the criteria of Bonferroni correction, *p*-values derived from the multiple comparisons were multiplied by the number of multiple comparisons (e.g., *p*-value × 2) to correct for false positives. Multiple logistic regression analysis was conducted to obtain the odds ratios (ORs) with 95 % confidence intervals (CIs) on the relative abundance of the II or II+ID genotypes within the overweight/obese group after adjusting for sex and age. All statistical analyses were conducted using SPSS Statistics software (Version 25, IBM Corporation, IL, USA) with *p*-value < 0.05 considered statistically significant.

## RESULTS

### Clinical characteristics among the Korean adults differentiated into normal, overweight and obese groups based on their BMI

The characteristics of individuals within the normal, overweight and obese groups are shown in Table 1. The groups were categorized based on the BMI criteria for Korean adults^31^. Among the participants, 103 (29.2 %) were classified as normal with a BMI < 23 kg/m^2^, 117 (33.1 %) were classified as overweight with a 23 kg/m^2^ ≤ BMI < 25 kg/m^2^, and 133 (37.7 %) were grouped as obese with a BMI ≥ 25 kg/m^2^. Anthropometric indexes, age and height were significantly higher in both overweight and obese groups compared to the normal group (*p*<0.05). Body weight and BMI were significantly different among all three groups, whereas PBF only differed between the obese and normal groups (*p*<0.05). SBP increased with the increase in fat storage. Additionally, obese individual had a greater DBP than in other groups (*p*<0.05). However, no relationship between pathogenic hypertension and the stratum of obesity was observed.

**Table 1.** Clinical characteristics of the normal, overweight and obese groups

Parameters	Normal (n=103)	Overweight (n=117)	Obese (n=133)
	BMI < 23	23 ≤ BMI < 25	BMI ≥ 25
Age (year)	55.3 ± 11.2^a^	59.2 ± 11.0^b^	58.2 ± 11.8^b^
Height (cm)	159.2 ± 6.8^a^	161.7 ± 8.1^b^	162.5 ± 8.7^b^
Body weight (kg)	54.3 ± 5.7^a^	62.9 ± 6.2^b^	71.5 ± 9.0^c^
PBF (%)	26.8 ± 6.6^a^	27.6 ± 6.3^a^	31.9 ± 7.0^b^
BMI (kg/m^2^)	21.4 ± 1.1^a^	24.0 ± 0.6^b^	27.0 ± 1.9^c^
SBP (mmHg)	124 ± 18^a^	129 ± 18^b^	134 ± 16^c^
DBP (mmHg)	75 ± 13^a^	75 ± 14^a^	80 ± 16^b^

Values are means ± SEM. PBF=percent body fat, BMI=body mass index,

SBP=systolic blood pressure, DBP=diastolic blood pressure.

Different superscripts (a, b, and c) represent significant differences at *p* < 0.05.

### *ACE* I/D polymorphism - mediated changes in the clinical phenotypes of Korean adults

The effect of *ACE* I/D polymorphism on clinically important parameters are presented in Table 2. Among the 353 participants, the II, ID and DD *ACE* genotypes were detected in 122, 177 and 54 individuals, respectively. This cluster of *ACE* genotypes did not differ from the expected Hardy-Weinberg equilibrium (*p*=0.607). Additionally, most variables including age, body weight, PBF, BMI, and even SBP/DBP were not significantly altered among the *ACE* genotypes. Contrastingly, the heights (cm) of participants who comprised of the ID (161.6 ± 8.2) and DD genotypes (163.7 ± 8.0) were taller than those with the II genotype (159.7 ± 7.8)(*p*<0.05).

**Table 2.** Characteristics of individuals grouped in accordance to their *ACE* genotypes

Parameters	*ACE* genotypes			HWE (*p*=0.607)
	II (n=122)	ID (n=177)	DD (n=54)	
Age (year)	60.0 ± 11.5	57.6 ± 11.4	57.2 ± 11.8	
Height (cm)	159.7 ± 7.8^a^	161.6 ± 8.2^b^	163.7 ± 8.0^b^	
Body weight (kg)	62.3 ± 9.4	64.2 ± 10.2	64.9 ± 10.9	
PBF (%)	29.9 ± 7.0	28.7 ± 6.9	27.8 ± 7.3	
BMI (kg/m^2^)	24.3 ± 2.5	24.5 ± 2.6	24.1 ± 3.1	
SBP (mmHg)	127 ± 16	131 ± 18	129 ± 17	
DBP (mmHg)	79 ± 9	76 ± 17	75 ± 14	

Values are means ± SEM. PBF=percent body fat, BMI=body mass index,

SBP=systolic blood pressure, DBP=diastolic blood pressure,

HWE=Hardy-Weinberg equilibrium.

Different superscripts (a and b) represent significant differences at *p* < 0.05.

### Association between *ACE* I/D polymorphism and overweight/obesity in Korean adults

The use of *ACE* I/D polymorphism as a potential genomic biomarker in the development of obesity is indicated in Table 3. The distribution of the *ACE* genotypes were not significantly different among the normal (II: n=38, 36.9%, ID: n=46, 36.8 %, DD: n=19, 18.4 %), overweight (II: n=43, 36.8%, ID: n=55, 47.0 %, DD: n=19, 16.2 %), and obese (II: n=41, 30.8%, ID: n=76, 57.0 %, DD: n=16, 12.0 %) groups. Unexpectedly, the I allele (normal: n=122, 48.8 %, overweight: n=141, 60.3 %, obese: n=158, 59.4 %) was preferred over the D allele (normal: n=84, 51.2 %, overweight: n=93, 39.7 %, obese: n=108, 40.6 %) in obese individuals. OR and 95 % CI were used to calculate the strength of association in the allele and genotype frequencies of the overweight and obese groups against the normal group, respectively.

**Table 3.** Genotype and allele frequencies of the *ACE* gene polymorphism among the normal, overweight and obese groups

Genotypes	Normal (n=103)	Overweight (n=117)	Obese (n=133)	Overweight vs Normal	Obese vs Normal
				OR (95% CI)	OR (95% CI)
II(n=122)	38(36.9)	43(36.8)	41(30.8)	-	-
ID(n=177)	46(36.8)	55(47.0)	76(57.1)	1.06(0.58~1.90)	1.13(0.63~2.04)
DD(n=54)	19(18.4)	19(16.2)	16(12.0)	0.88(0.49~1.91)	0.78(0.35~1.73)
II+ID(vs. DD)	84(81.6)	98(83.8)	117(88.0)	0.83(0.41~1.67)	0.58(0.28~1.20)
I allele	122(48.8)	141(60.3)	158(59.4)		
D allele	84(51.2)	93(39.7)	108(40.6)		

Values are shown as number (%) or OR (95% CI).

OR=odds ratio, CI=confidence interval.

A greater proportion of participants with the ID genotype were intended to being overweight (OR: 1.06, 95% CI: 0.58~1.90) and obese (OR: 1.13, 95% CI: 0.63~2.04), compared to the lower proportion of participants with the DD genotype was likely to being overweight (OR: 0.88, 95% CI: 0.49~1.91) and obese (OR: 0.78, 95% CI: 0.35~1.73). Besides, the newly generated II+ID genotype appears to be associated with a greater proportion of individuals being overweight (OR: 0.83, 95% CI: 0.41~1.67) and obese (OR: 0.58, 95% CI: 0.28~1.20) compared to the normal. Thus, the data suggests that the ACE I/D polymorphism does not affect the risk of obesity in Korean adults.

## DISCUSSION

The present study is the first to investigate the association between the *ACE* I/D polymorphism and susceptibility for overweight/obesity of healthy Korean adults. The data obtained from the adult population was not subdivided into age and sex to ensure a massive and representative cluster so that any negative impact of selection bias can be eliminated.

Overweight/obesity is a multifactorial disease that is frequently associated with comorbidities including chronic diseases. As a result of the rising prevalence, morbidity and mortality rates of obesity, prevention strategies remain an essential focus. Hence, it is imperative to determine if the gene-environment interactions exacerbate the genotype-phenotype relationship.

Considering the role of local RAS in regulating obesity, Ang II, a potent vasoconstrictor, promotes lipogenesis in adipocytes^33^. Moreover, genetic variants of *ACE* have been implicated in adipogenesis and metabolic disease^34-36^. Therefore, the association between *ACE* I/D polymorphism and obesity is plausible, as illustrated in several studies^21-23^.

The observations made in this study are as follows: (i) hypertension was not a pathological occurrence of obesity; However, BP (SBP and DBP) was higher in obese (BMI ≥ 25) individuals compared to normal. (ii) *ACE* genotypes do not have any impact on obesity and hypertension. Previously, Um et al^22^ also found no association between *ACE* I/D polymorphism and obesity in Korean women. Moreover, Thomas et al^37^ proposed that pathological hypertension is not associated with *ACE* I/D polymorphism. (iii) ID genotype is proposed to be the most vulnerable to the development of obesity compared to the DD and ID+DD genotype; However, it’s association with obesity is not statistically favorable. Contrary to this findings, previous studies have shown a positive association between *ACE* I/D polymorphism and obesity, as the D allele of the ACE gene is frequently detected in younger obese men including preschool children, children and adolescents^5,28^. Furthermore, a recent meta-analysis study suggested that the DD genotype is related to a higher susceptibility for overweight/obesity in an overall population, especially in Africans^8^. BMI is closely associated with the overall subcutaneous fat mass but is a poor predictor of central obesity (abdominal and visceral) when compared to waist circumference. Thus, further studies using the gold-standard measurement for body composition are required, with a large sample-size in both the adults and elderly populations. Moreover, a genetic predisposition to obesity primarily focusses on single nucleotide polymorphisms (SNPs); However, environmental and epigenetic factors have been recently recognized to be contributors for the development of obesity^38^. The limitations of this study include the potential contribution of confounding factors (obesity-related blood parameters and a history of medications). However, this study can be used to support further studies in Korean clusters.

In conclusion, *ACE* I/D polymorphism has not been observed to be a powerful candidate gene for obesity in Korean adults.

